# Fear of Movement/(Re)Injury: An Update to Descriptive Review of the Related Measures

**DOI:** 10.3389/fpsyg.2021.696762

**Published:** 2021-07-07

**Authors:** Haowei Liu, Li Huang, Zongqian Yang, Hansen Li, Zhenhuan Wang, Li Peng

**Affiliations:** ^1^College of Physical Education, Southwest University, Chongqing, China; ^2^Key Lab of Physical Fitness Evaluation and Motor Function Monitoring, Southwest University, Chongqing, China

**Keywords:** fear of movement/(re)injury, kinesiophobia, scales, questionnaires, scoring system

## Abstract

The prevalence of fear of movement (kinesiophobia) in persistent pain ranges from 50 to 70%, and it may hinder the subsequent rehabilitation interventions. Therefore, the evaluation of fear of movement/(re)injury plays a crucial role in making clinical treatment decisions conducive to the promotion of rehabilitation and prognosis. In the decision-making process of pain treatment, the assessment of fear of movement/(re)injury is mainly completed by scale/questionnaire. Scale/questionnaire is the most widely used instrument for measuring fear of movement/(re)injury in the decision-making process of pain treatment. At present, the most commonly used scale/questionnaire are the Tampa Scale for Kinesiophobia (TSK), the Fear-Avoidance Beliefs Questionnaire (FABQ), the Kinesiophobia Causes Scale (KCS), the Athlete Fear-Avoidance Questionnaire (AFAQ), and the Fear-Avoidance Components Scale (FACS). In order to provide necessary tools and references for related research and rehabilitation treatment, this descriptive review is designed as an introduction to the background and content, score system, available language versions, variants of the original questionnaire, and psychometric properties of these scales/questionnaries.

## Introduction

In the past several decades, the relation between fear and pain has been described with various constructs. Fear of movement/(re)injury, pain-related fear, fear-avoidance beliefs, and kinesiophobia are the most commonly used constructs (Lundberg et al., 2011). In the 1980s, Lethem et al. (1983) developed the fear-avoidance model (FAM) to explain why some injuries transform from acute to chronic while others heal in normal time frames. In 1995, Vlaeyen et al. (1995) expanded the FAM into the cognitive-behavioral model of fear of movement/(re)injury. In that model, fear of movement/(re)injury was considered an important factor for disability, disuse syndrome, and depression in patients with musculoskeletal pain. If the patients misinterpret the pain and magnify the condition, in that case, they are likely to enter a maladaptive cycle of where fear of pain occurs, which leads to avoidance behavior and fear of movement/(re)injury. During the development of FAM, Kori (1990) proposed the concept of kinesiophobia that was defined for a patient who has “an excessive, irrational, and debilitating fear of physical movement and activity resulting from a feeling of vulnerability to painful injury or reinjury.” The prevalence of kinesiophobia in persistent pain ranges from 50 to 70% (Luque-Suarez et al., 2019). A considerable number of longitudinal studies have found that a high level of kinesophobia at baseline can be used to predict the decreased quality of life, increased pain, and disability (Wong et al., 2015; Helminen et al., 2016; Maaike et al., 2017). Initially, the concept of “fear of movement/(re)injury” was applied to patients with chronic musculoskeletal pain (Vlaeyen et al., 1995). However, with the continuous exploration of researchers worldwide, research on other consequences accompanied by fear of movement/(re)injury gradually increased. In the clinical setting, fear of movement/(re)injury is an essential factor affecting the surgery (Doménech et al., 2014) and prognosis of physical therapy (Verwoerd et al., 2015), and it also can hinder the subsequent recovery of physical activity (Boutevillain et al., 2017). Assessing the fear of movement/(re)injury of the patients is helpful for researchers to explore the mechanism of chronic musculoskeletal pain more deeply and is useful for clinicians to make better clinical decisions.

Extensive research has shown that fear of movement/(re)injury is usually assessed with scales/questionnaires. The Tampa Scale for Kinesiophobia (TSK) (Miller et al., 1991) and the Fear-Avoidance Beliefs Questionnaire (FABQ) (Waddell et al., 1993) were designed in the early years. Some new measures like the Kinesiophobia Causes Scale (KCS) (Knapik et al., 2011), Athlete Fear-avoidance Questionnaire (AFAQ) (Dover and Amar, 2015), and Fear-Avoidance Components Scale (FACS) (Neblett et al., 2016) have been developed in recent years. Thus, renewing the information of these instruments is necessary for researchers and clinicians. The purpose of this article is to provide a descriptive review of each measure regarding the background and content, score system, available language versions, variants of the original questionnaire, and psychometric properties, which can offer the basis of scale selection and application reference for the research and clinical treatment of fear of movement/(re)injury.

## Methodology

A search procedure, which involved searching electronic databases such as PubMed (https://pubmed.ncbi.nlm.nih.gov/), Web of Science (http://isiknowledge.com/), and Google Scholar (https://scholar.google.com/), was developed. The databases were searched from 1980 to 2021. Searches were carried out for the terms “kinesiophobia,” “fear of movement/(re)injury,” “scales,” “questionnaires,” “sports,” and “fear-avoidance.” All references were downloaded into the Zotero (version 5.0.96.2), which facilitated the large number of publications obtained. The aim of this review was to ensure that all the essential published papers were identified; nevertheless, some may have been missed out from the present review.

## The Tampa Scale for Kinesiophobia

### Background and Content

Miller et al. (1991) designed the TSK in 1991, but it was not published until 1995 (Acar et al., 2016). The TSK is one of the most well-known instruments for measuring fear of movement/(re)injury. Different models were proposed based on exploratory factor analysis (EFA) and confirmatory factor analysis (CFA): the generally accepted 2-factor model labeled somatic focus (TSK-SF; beliefs in underlying and serious medical problems) and activity avoidance (TSK-AA; beliefs that activity may result in [re]injury or increased pain) (Roelofs et al., 2004). Item response on a 5-point Likert scale range from 0 (strongly agree) to 4 (strongly disagree), including statements such as “I'm afraid that I might injure myself if I exercise (item 1)” and “If I were to try to overcome it, my pain would increase (item 2).”

### Score System

The original TSK includes 17 items, among which 4 items (i.e., items 4, 8, 12, and 16) are reverse-scored. The test's total score is the sum of the points marked by the patient, ranging from 17 to 68. The higher the score, the higher the fear of movement/(re)injury of the patient. If the score is >37, the patient is considered to suffer from kinesiophobia.

### Available Language Versions

As far as we know, it has been translated into Italian (Monticone et al., 2010), Japanese (Huang et al., 2019), Swedish (Larsson et al., 2014), Turkish (Acar et al., 2016), Dutch (Visscher et al., 2010), Chinese (Cai et al., 2019), Spanish (Aguiar et al., 2017), and Norwegian (Haugen et al., 2008).

### Variants of the Original Questionnaire

Except low back pain, for which it was originally applied, the TSK was adapted for ACL injuries (Luc-Harkey et al., 2018; Huang et al., 2019), heart failure and pulmonary arterial hypertension (Acar et al., 2016), and temporomandibular disorders (Garrigós-Pedrón et al., 2018). The shortened versions of the TSK are TSK-13 (Jørgensen et al., 2015), TSK-12 (Visscher et al., 2010), TSK-11 (Kikuchi et al., 2015; Goldberg et al., 2018), and TSK-4 (Gregg et al., 2015), among which TSK-11 is the most widely used.

### Psychometric Properties

The Cronbach's α of each version of the TSK scale is generally between 0.7 and 0.92, and the test-retest reliability is generally above 0.8 (Swinkels-Meewisse et al., 2003; Woby et al., 2005; Huang et al., 2019). Moderate correlation coefficients supported the construct validity with pain-related fear, pain catastrophizing, and disability in patients with chronic low back pain. Correlation coefficients supported a moderate predictive validity with performance on physical performance tests in patients with chronic low back pain. Concurrent validity is moderate, supported by Pearson's rho between TSK and FABQ in patients with acute low back pain, ranging from *r* = 0.33 to 0.59 (*P* < 0.01; Swinkels-Meewisse et al., 2003).

## Fear-Avoidance Beliefs Questionnaire

### Background and Content

The FABQ is a patient-reported questionnaire specially designed to measure fear-avoidance beliefs of patients about physical activity and work. It was developed by Waddell and published in 1993 (Waddell et al., 1993). The FABQ is a questionnaire based on the FAM, which was created to explain why some patients with acute painful conditions can recover while others develop chronic pain from such conditions (Lethem et al., 1983; Fritz and George, 2002). The FABQ total (FABQ-T) includes two subscales, the work subscale (FABQ-W) and the physical activity subscale (FABQ-PA). The form of answer is a standardized option (7-point Likert fields), and each question is scored ranging from “completely disagree” (0) to “completely agree” (6). CFA showed that item 1 had low communality and had inconsistent factor loading while items 13, 14, and 16 were redundant (Waddell et al., 1993).

### Score System

There are 16 items within the FABQ (maximum score of 66); FABQ-W with 7 questions (maximum score of 42) and FABQ-PA with 4 questions (maximum score of 24). Users should note that items 1, 8, 13, 14, and 16 are not scored (Table 1).

**Table 1:** The items, total possible points, and high score from the fear-avoidance beliefs questionnaire (FABQ) and its subscale.

**Scale**	**Questions included**	**Total possible points**	**High score**
FABQ-T	2–7, 9–12, 15	66	None
FABQ-W	6, 7, 9–12, 15	42	>34 Fritz and George, 2002
FABQ-PA	2–5	24	>15 Crombez et al., 1999

### Available Language Versions

So far, in terms of the literature that we can be searched, the FABQ has been translated into Chinese (Pei et al., 2010), German (Pfingsten, 2004), Italian (Meroni et al., 2014), Brazilian (Abreu et al., 2008), Greek (Georgoudis et al., 2007), Thai (Wiangkham et al., 2020), Finnish (Pfingsten, 2016), and Hausa (Brox, 2019) d.

### Variants of the Original Questionnaire

The FABQ has been adapted to assess fear-avoidance beliefs of patients in multiple areas, including low back pain (Fujii et al., 2013), chronic headache (Nash et al., 2006), fibromyalgia (Roelofs et al., 2004), neck pain (Lee et al., 2006), knee pain (Ross, 2010), shoulder pain (Mintken et al., 2010), osteoarthritis (Heuts et al., 2004), and even extended to burning pain (Sgroi et al., 2005) and complex regional pain syndrome type I (Jong et al., 2005).

### Psychometric Properties

Most studies have verified the excellent reliability of the FABQ. A study showed that the test-retest reliability of FABQ-T, FABQ-W, and FABQ-PA are 0.97, 0.72 ~ 0.90, and 0.80 ~ 0.91, respectively (Williamson, 2006). Abreu et al. (2008) validated the Portuguese version of the FABQ the reliability of patients with low back pain, which showed that FABQ-P (ICC = 0.84, Cronbach's α = 0.80) and FABQ-W (ICC = 0.91, Cronbach's α = 0.90). The correlation coefficients of FABQ-T, FABQ-W, and FABQ-PA with Roland and Morris Disability Questionnaire (RMDQ) are 0.52, 0.63, and 0.51, respectively (Williamson, 2006). The correlation between TSK and FABQ-W and FABQ-PA was 0.33 and 0.39 (Swinkels-Meewisse et al., 2003). These pieces of evidence mentioned above supported that FABQ has good criterion validity. However, a study reported that the structural validity of FABQ was relatively low and that there was little evidence about the responsiveness before and after treatment (Lundberg et al., 2011).

## The Kinesiophobia Causes Scale

### Background and Content

The KCS is a relatively new patient-reported outcome measure used to identify the biological and psychological causes of kinesiophobia in people (Knapik et al., 2011).

### Score System

This scale is composed of 20 closed questions. The domain score is the mean of the total factors that make up the domain, while the overall index of kinesiophobia (KCS) is the mean of two domains. According to Knapik's assumption, the total KCS score will range from 0 to 100 and can be interpreted as a percent of kinesiophobic behavior—a higher score indicating higher fear of movement (Knapik et al., 2011). The calculations of the biological domain, psychological domain, and total KCS score are performed as (A + B + C + D)/4, (E + F + G + H)/4, and (Biological Domain + Psychological Domain)/2, respectively (Table 2).

**Table 2:** The domain, dimensions, and calculation of the kinesiophobia causes scale (KCS).

**Domain**	**Dimension**	**Calculations**
Biological domain	A. Morphologic (items 1–2) B. Individual need for stimulation (items 3–5) C. Energetic substrates (items 6-9) D. Power of biological drives (items 10-11)	A = items (1 + 2)/2 B = items (3 + 4+5)/3 C = items (6 + 7 + 8 + 9)/4 D = items (10 + 11)/2
Psychological domain	E. Self-Acceptance (items 12–14) F. Self-Assessment of motor predispositions (items 15–16) G. State of mind (items 17–18) H. Susceptibility to social influence (items 19–20)	E = items (12 + 13 + 14)/3 F = items (15 + 16)/2 G = items (17 + 18)/2 H = items (19 + 20)/2

### Available Language Versions

Currently, as far as the literature can be searched, English version (Knapik et al., 2011), Polish version (Brdak et al., 2015), Turkish version (Çayir et al., 2020), and Chinese version (Zhu et al., 2020) are available.

### Variants of the Original Questionnaire

No other variants are available.

### Psychometric Properties

The KCS was characterized with good internal consistency in a few studies. Saulicz et al. (2016) used the KCS to evaluate 105 women of perimenopausal age and verified that KCS had good internal consistency. Cronbach's α of the biological and psychological domain subscale were 0.79 and 0.77, respectively. The Cronbach's α of KCS total scale, biological domain, and psychological domain in the Turkish version are 0.86, 0.91, and 0.80, respectively (Çayir et al., 2020).

## Athlete Fear-Avoidance Questionnaire

### Background and Content

In 2015, Dover developed the AFAQ, a sport-specific scale, to identify the high levels of fear-avoidance in athletes (Dover and Amar, 2015). Therapists and trainers can use it as a tool to address this psychological barrier early in rehabilitation and potentially reduce the time until they return to the game. As it is specially developed for athletes, the scale uses relevant terms that athletes can understand, including the expressions of “I will never be able to play as I did before the injury (item 1)” and “I believe that my current injury has jeopardized my future athletic abilities (item 5).” The AFAQ contains 10 items related to sports psychology, sports injury, and sports experience. The scale is measured on a 5-point Likert scale ranging from 1 (Not at all) to 5 (completely agree).

### Score System

The total score ranged from 10 to 50, where the higher the score, the more fear-avoidance the athletes possess (Dover and Amar, 2015).

### Available Language Versions

To our knowledge, the scale is available in three languages, English (Dover and Amar, 2015), Japanese (Fukano et al., 2019), and Portuguese (Leitão, 2019).

### Variants of the Original Questionnaire

O'Keeffe et al. (2020) developed a modified AFAQ (mAFAQ) to make it a screening tool for fear-avoidance of athletes. Unlike the original version, the sentence “if I am injured” is added to each of the 10 statements to predict the degree of injury-related fear-avoidance that an athlete may occur after injury. The study showed that mAFAQ is a valid and reliable screening tool in predicting injury (O'Keeffe et al., 2020).

### Psychometric Properties

The internal consistency of AFAQ was very high (Cronbach's α = 0.805), and it was significantly correlated with FABQ and other assessment tools (*r* = 0.352, *P* < 001), which verified its concurrent validity (Dover and Amar, 2015). The results of the Portuguese version showed that the test-retest reliability of AFAQ is excellent (ICC = 0.969) (Leitão, 2019).

## Fear-Avoidance Components Scale

### Background and Content

The FACS is a newly developed scale developed by Neblett et al. (2016) in 2016, which combines the essential components of several well-studied scales (TSK, FABQ, Pain Anxiety Symptom Scale, and Pain Catastrophizing Scale), to evaluate the psychological characteristics of fear-avoidance in patients with painful medical conditions comprehensively. The items of the FACS are trying to correct the deficiencies of the above scales based on the latest fear-avoidance model. There were 20 items in the FACS, and each item was scored on a 6-point Likert scale ranging from 0 (completely disagree) to 5 (completely agree). The total score was 0–100, indicating subclinical (0–20), mild (21–40), moderate (41–60), severe (61–80), and extreme (81–100).

### Score System

The final score is the sum of each item. Higher scores are intended to indicate higher levels of fear-avoidance (Neblett et al., 2016).

### Available Language Versions

So far, there are four language versions available, including English (Neblett et al., 2016), Serbian (Knezevic et al., 2018), Gujarati (Bid et al., 2020), and Spanish (Cuesta-Vargas et al., 2020).

### Variants of the Original Questionnaire

No other variants are available.

### Psychometric Properties

Tested by Neblett et al. (2016), the English version of FACS has good internal consistency (Chronbach's α = 0.92) and high test-retest reliability (*r* = 0.90 ~ 0.94, *P* < 0.01). In 2018, the Serbian version of FACS introduced by Knezevic et al. (2018) studied 322 patients with chronic musculoskeletal pain. In 2020, Cuesta-Vargas et al. (2020) selected 330 patients with chronic musculoskeletal pain and verified the adaptability of the Spanish version (FACS-Sp). Their Cronbach's α were 0.90 and 0.88, respectively. The convergent validity is supported by Pearson's correlation with Central Sensitization Inventory (*r* = 0.414).

## Discussion

The scales and questionnaires determine the severity of fear of movement/(re)injury in patients with different medical conditions. By presenting these five scales, it can be seen that the assessments of fear of movement/(re)injury tend to be more rigorously subdivided, such as special populations, causes, and components, and are becoming more comprehensive. From a perspective of use, the citations from high to low were FABQ (3268), TSK (493), KCS (61), AFAQ (29), and FACS (26). It is noted that researchers or clinicians need to be cautious in selecting the last three tables, due to which they were not well-studied as the former two. The TSK is the earliest instrument to measure fear of movement/(re)injury, and it has many language versions and a wide range of applications. A study showed that the TSK was sensitive in detecting clinical changes in subjects undergoing rehabilitation after lumbar fusion and chronic low back pain (Monticone et al., 2016). However, the validity of the TSK was low to moderate (Lundberg et al., 2011). Compared with the other shortened versions, the internal consistency of TSK-4 is insufficient (Archer et al., 2012). According to Japanese research on the TSK, the scale may not be the best tool for assessing psychological factors in patients with knee anterior ligament injury (Huang et al., 2019). Currently, most studies believed the FABQ seems to be the best available measure to measure “fear-avoidance beliefs.” Still, a recent study questioned this, suggesting that the FABQ questionnaire is most likely related to expectations rather than fear (Aasdahl et al., 2020). The FABQ can discriminate between patients with cervical radiculopathy and healthy subjects (Dedering and Börjesson, 2013). Compared with the TSK, a recognized cutoff score of the FABQ is still not available (Wertli et al., 2014). In addition, the construct validity of FABQ is relatively low, and there is little evidence of responsiveness before and after treatment (Lundberg et al., 2011). The KCS tries to identify and quantify the causes of kinesiophobia in patients from two domains: biology and psychology. Nevertheless, the research on KCS is dominated by Polish researchers, and the cross-cultural adaptation and validation of other versions need to be further studied. Moreover, the KCS scoring system is a little bit complicated and requires special attention when using it. The AFAQ is a sport-specific questionnaire used to evaluate the thoughts of athletes regarding injury and return to the competitions. However, there are few studies on the reliability and validity of AFAQ. Whether the questionnaire is still reliable in other languages/cultural backgrounds needs more studies to verify. The FACS is based on a developed FAM to improve the disadvantage of the well-studied measures. It has good psychometric characteristics and has five grades of severity range for clinical interpretation. It seems the most comprehensive scale so far. The application of FACS is still few, and its characteristics and limitations need to be further explored.

## Conclusion

The present review has provided a general description of existing measures from 1991 (TSK) to 2016 (FACS). These scales/questionnaires mentioned above are useful tools for assessing the constructs related to fear of movement/(re)injury in several different types of studies. After comparison, it was found that no scale/questionnaire can evaluate all the characteristics of fear of movement/(re)injury. Still, the results of the measurements illustrate how much the different aspects of fear of movement/(re)injury limit the ability of the patient to perform the necessary life functions, thus giving an idea of how it affects the quality of life, pain, and disability. Considering that fear of movement/(re)injury has become a common factor of rehabilitation, using the scales presented allows clinicians to assess surgical/rehabilitation treatment results. Each questionnaire should be considered for its characteristics when using in research and clinical practice. If necessary, the combination of those scales can be considered. Therefore, with the help of adequately used fear of movement/(re)injury outcome measures, effective treatment methods can be selected and applied.

## Author Contributions

HLiu composed this study. LH designed the framework. ZY, HLi, and ZW revised the manuscript. LP provided supervision throughout the research and made critical revisions to this study. All authors contributed to the article and approved the submitted version.

## Conflict of Interest

The authors declare that the research was conducted in the absence of any commercial or financial relationships that could be construed as a potential conflict of interest.
