# Supplementary material for: Fear of Movement/(Re)Injury: An Update to Descriptive Review of the Related Measures
Source: Front Psychol. 2021 Jul 7;12:696762. doi: 10.3389/fpsyg.2021.696762 (PMC8292789; doi:10.3389/fpsyg.2021.696762)
Supplement: Supplementary file 1 [file Table_1.DOCX]

Supplementary Material

**1. Tampa Scales for Kinesiophobia**

| Item and description | |
| --- | --- |
| 1 | I am afraid that I might injure myself if I exercise |
| 2 | If I were to try to overcome it, my pain would increase |
| 3 | My body is telling me I have something dangerously wrong |
| 4 | My pain would probably be relieved if I were to exercise |
| 5 | People are not taking my medical condition seriously enough |
| 6 | My accident has put my body at risk for the rest of my life |
| 7 | Pain always means I have injured my body |
| 8 | Just because something aggravates my pain does not mean it is dangerous |
| 9 | I am afraid that I might injure myself accidentally |
| 10 | Simply being careful that I do not make any unnecessary movements is the safest thing I can do to prevent my pain from worsening |
| 11 | I would not have this much pain if there were not something potentially dangerous going on in my body |
| 12 | Although my condition is painful, I would be better off if I were physically active |
| 13 | Pain lets me know when to stop exercising so that I do not injure myself |
| 14 | It is really not safe for a person with a condition like mine to be physically active |
| 15 | I can not do all the things normal people do because it is too easy for me to get injured |
| 16 | Even though something is causing me a lot of pain, I do not think it is actually dangerous |
| 17 | No one should have to exercise when he/she is in pain |

Items 1, 2, 3, 5, 6, 7, 10, 11, 13, 15, and 17 are TSK-11 items

**2. Fear-Avoidance Beliefs Questionnaire**

**Here are some of the things which other patients have told us about their pain. For each statement please circle any number from 0 to 6 to say how much physical activities such as bending, lifting, walking or driving affect or would affect your back pain.**

| Item and description | |
| --- | --- |
| 1 | My pain was caused by physical activity |
| 2 | Physical activity makes my pain worse |
| 3 | physical activity might harm my back |
| 4 | I should not do physical activities which (might) make my pain worse |
| 5 | I cannot do physical activities which (might) make my pain worse |
| 6 | My pain was caused by my work or by an accident at work |
| 7 | My work aggravated my pain |
| 8 | I have a claim for compensation for my pain |
| 9 | My work is too heavy for me |
| 10 | My work makes or would make my pain worse |
| 11 | My work might harm my back |
| 12 | I should not do my normal work with my present pain |
| 13 | I cannot do my normal work with my present pain |
| 14 | I cannot do my normal work till my pain is treated |
| 15 | I do not think that I will be back to my normal work within 3 months |
| 16 | I do not think that I will ever be able to go back to that work |

**3. Kinesiophobia Causes Scale**

**Dear Madam/Dear Sir**

**You are kindly requested to choose ONE answer for EACH item below except items 8 and 13 where you will choose between yes/no/not sure for EACH answer**

**1. According to my body mass, I can claim that:**

a) I control my body mass adjusting the level of motor activity [0]

b) my body mass causes difficulties in performing several motor tasks, so I avoid them [50]

c) due to overweight, I avoid physical efforts because of the risk of exhaustion or injury [100]

**2. I feel that because of its shape, my body causes motor limitations in sport activities I would like to perform:**

a) almost never [0]

b) seldom [25]

c) sometimes [50]

d) often [75]

e) very often [100]

**3. I think that in comparison with others I am always perceived as:**

a) less active [100]

b) less active than people of my age [75]

c) equally active as people of my age [50]

d) more active than other people [25]

e) far more active than other people [0]

**4. Prolonged sitting:**

a) feels pleasant to me, I can assume and maintain such position for a long time [100]

b) similarly to other people, when it lasts too long, I have to change position [50]

c) seems uncomfortable to me, I avoid prolonged sitting [0]

**5. While working, I try to find a way demanding the least physical effort because I do not like physical fatigue:**

a) always [100]

b) sometimes [50]

c) never [0]

**6. I believe that activities demanding intensive physical effort:**

a) are fatiguing to me and I try to avoid them if possible [100]

b) are possible, it depends what specifically I should do [50]

c) give me pleasure because physical fatigue means satisfaction to me [0]

**7. When I am physically exhausted:**

a) I feel bad and it takes long time to recover [100]

b) I recover as quickly as other people of my age [50]

c) I recover quickly and I feel energy to start new actions [0]

**8. I believe that irrespectively of my present state of mind I could with NO rest:**

yes not sure no

1. walk for 1 hour [0] [50] [100]

b) climb third floor [0] [50] [100]

c) ride a bike for 0.5 hour [0] [50] [100]

**9. After work, I usually feel:**

a) tired, but after a little rest, I am ready to start activity (housework, visiting friends, going to the cinema, theatre, walking or sport) [0]

b) tired and I rest passively either lying or sitting [50]

c) rather exhausted than tired, and I always rest for a long time either lying or sitting [100]

**10. Competition in sport, work, etc.:**

a) always makes me satisfied and gives opportunity to win [0]

b) is acceptable in disciplines I feel good at, then I like to compete [50]

c) is out of question, I’m very sensitive to failures [100]

**11. I feel irritated when** **circumstances force me**

a) always [100]

b) often [75]

c) sometimes [50]

d) seldom [25]

e) never [0]

**12. In relation to my own appearance:**

a) I never felt embarrassed by the shape of my body. Wearing clothes exposing it (e.g. sport clothes or) swimsuit do not seem problematic to me irrespective of how do other people look like [0]

b) I can wear sport or swimsuit on the condition that people around look similarly [50]

c) I avoid situations in which clothing would expose shortcomings of my figure  [100]

**13. I believe that activities mentioned below should, because of cultural reasons, match age and/or social status of a given individual:**

yes no

a) dancing [100] [0]

b) sport [100] [0]

c) fatiguing non-profit tasks (e.g. housework, gardening, DIY) [100] [0]

**14. At the opportunity of participation in sport (holidays, encouragement from other people):**

a) I always try to use it [100]

b) I feel certain resistance, but usually I agree [75]

c) first I watch others and try to judge my chances for good performance and then I take my decisions [50]

d) it is very difficult to convince me, I rarely agree [25]

e) no, this is not for me [0]

**15. In comparison with other people, I believe that I can learn new movements (motor skills):**

a) more quickly than others [0]

b) more quickly than people of my age [25]

c) as quickly as people of my age  [50]

d) more slowly than others [75]

e) I cannot learn any motor skill  [100]

**16. During my childhood and adolescence:**

a) I did not participate in sport (only obligatory exercises) [100]

b) I participated in sport as often as other kids [50]

c) I was more active than others (e.g. training in a sport club) [0]

**17. Considering pain, trauma and injuries:**

a) I believe that in life, there is always a risk of sickness and injury, but this is not a factor reducing my motor activity [0]

b) I believe that it is necessary to act in accordance to the “common sense” and adjust the level and type of activity to an individual’s age and abilities [50]

c) I believe that increased activity may be harmful, special care should be taken [100]

**18. When I become sick or sustain an injury, I believe that:**

a) first is to recover completely and then to start regular activity [100]

b) reasonable level of motor activity is necessary, in accordance to medical indications and my

own condition [50]

c) frequently the best way to fight the problem is to ignore the pain and lead normal, active life [0]

**19. In comparison with my relatives, friends and mates:**

a) I rest more actively than they do [0]

b) I rest typically for my age and gender [50]

c) I rest more passively than they do  [100]

**20. In comparison with other expenses, expenses on active recreation are for me:**

a) less important [100]

b) equally important [50]

c) more important [0]

**4. Athlete Fear Avoidance Questionnaire**

**Instructions: We are interested in your feelings or thoughts when in pain as a result of a sport injury. Using the following scale, please indicate the degree to which you have these thoughts and feelings when you are in pain due to a sports injury.**

| Item and description | |
| --- | --- |
| 1 | I will never be able to play as I did before my injury |
| 2 | I am worried about my role with the team changing |
| 3 | I am worried about what other people will think of me if I don't perform at the same level |
| 4 | I am not sure what my injury is |
| 5 | I believe that my current injury has jeopardized my future athletic abilities |
| 6 | I am not comfortable going back to play until I am 100% |
| 7 | People don't understand how serious my injury is |
| 8 | I don't know if I am ready to play |
| 9 | I worry if I go back to play too soon I will make my injury worse |
| 10 | When my pain is intense, I worry that my injury is a very serious one |

**5. Fear-avoidance Components Scale**

**Instructions: People respond to pain in different ways. We want to find out how you think and feel about your painful medical condition and how it has affected your activity level. Please think about how you have been over the past week, and circle one number between “0” and “5”from the scale below to answer each question.**

| Item and description | |
| --- | --- |
| Over the past week, how much do you agree with these statements about your painful medical condition? | |
| 1 | I try to avoid activities and movements that make my pain worse  pain worse... |
| 2 | I worry about my painful medical condition |
| 3 | I believe that my pain will keep getting worse until I won't be able to function at all  won't be able to function at all.. |
| 4 | I am overwhelmed by fear when I think about my painful medical condition  medical condition. . |
| 5 | I don't attempt certain activities because I am fearful that I will injure (or re-injure) myself.  I will injure (or re-injure) myself. |
| 6 | When my pain is really bad, I also have other symptoms such as nausea, difficulty breathing, heart pounding, trembling, and/or dizziness  such as nausea, difficulty breathing, heart pounding,  trembling,and/or dizziness.......... |
| 7 | It is unfair that I have to live with my painful medical condition  condition.. ................. |
| 8 | My painful medical condition puts me at risk for future injuries (or re-injuries) for the rest of my life  injuries (or re-injuries) for the rest of my life.... |
| Over the past week, how much do you agree with these statements about your painful medical condition? | |
| 9 | Because of my painful medical condition, my life will never be the same  never be the same........... |
| 10 | I have no control over my pain |
| 11 | I don't attempt certain activities and movements because I am fearful that my pain will increase  I am fearful that my pain will increase. . . . . . . .. . .. . |
| 12 | It is someone else's fault that I have this painful medical condition  medical condition.. |
| 13 | The pain from my medical condition is a warning signal that something is dangerously wrong with me  that something is dangerously wrong with me.......... |
| 14 | No one understands how severe my painful medical condition is  condition is.. |
| Start each of the following items with this statement: Over the past week, due to my painful medical condition I have avoided the following...  Over the past week, due to my painful medical condition I have avoided the following... | |
| 15 | …strenuous activities (like doing heavy yard work or moving heavy furniture)  moving heavy furniture)..................... |
| 16 | ...moderate activities (like cooking dinner or cleaning the house)  the house)....................... |
| 17 | ...light activities (like going to the movies or going out to lunch)  to lunch).................. |
| 18 | ...my full duties and chores at home and/or at work |
| 19 | ...recreation and/or exercise (things that I do for fun and good health)  good health)...................... |
| 20 | ...activities where I have to use my painful body part(s) |
